# Cyclophosphamide-induced testicular toxicity ameliorate by *American ginseng* treatment: An experimental study

**Published:** 2018-11

**Authors:** Akram Hosseini, Samad Zare, Zahra Borzouei, Firouz Ghaderi Pakdel

**Affiliations:** 1 *Research and Clinical Center for Infertility, Yazd Reproductive Sciences Institute, Shahid Sadoughi University of Medical Sciences, Yazd, Iran.*; 2 *Department of Biology, Faculty of Science, University of Urmia, * *Urmia, Iran* *.*; 3 *Solid Tumor Research Center Urmia* *.* * University of Medical Sciences, Urmia, Iran.*; 4 *Department of Physiology, Faculty of Medicine, Urmia University of Medical Sciences, Urmia, Iran. *

**Keywords:** Cyclophosphamide, American ginseng, Oxidative stress, DNA damage

## Abstract

**Background:**

Cyclophosphamide (CP) is one of the most invasive chemotherapeutic agents, which used commonly despite of its wide spectrum toxicity. Clinical evidence showed toxic side effects of CP in multiple organ systems.

**Objective:**

The objective of the present study was to evaluate the effects of *American ginseng* on CP-induced testicular toxicity in rats.

**Materials and Methods:**

Adult male Wistar rats (220±30 gr) were randomly divided into four groups (n=7 in each). Group 1 as control received normal saline by gavage, group 2 received CP (6.1 mg/kg/day, i.p.) for a period of 50 days. Group 3 received *American ginseng* (500 mg/kg/day) by gavage. Group 4 received *American ginseng *(500 mg/kg/day) 1h prior to the administration of CP in the equal dose of group 2. The animals scarified one day after the last treatment and the effects of *American ginseng* on the sperm vital parameters, testicular functions, biochemical factors, and structural malformations evaluated.

**Results:**

Serum testosterone concentration was significantly decreased whereas the level of malondialdehyde and DNA damage were significantly increased in animals of CP group (p<0.01). Co-administration of *American ginseng* reversed these parameters and improved recovery in CP+ginseng group. In addition, seminiferous tubules of testis severely damaged in the CP group but ginseng improved histologic changes in CP+ginseng group.

**Conclusion:**

The findings confirmed the protective effects of *American ginseng* on toxicity induced by CP in the reproductive system of male rats.

## Introduction

The failure to conceive after 1 yr of unprotected intercourse defined as infertility that affected about 15% of all couples in the world. There are some evidence that male infertility factors contribute to approximately half of all cases (1). The male infertility caused by many factors and may be attributed to intra- and/or extra-testicular factors (2). Most of these factors can induce alterations in sperm parameters. Low sperm concentration, poor sperm motility, abnormal sperm morphology, and impairment in DNA and chromatin integrity are the most alterations that produce (3). There are some other factors can produce infertility disorders such as: drugs, chemotherapy, radiotherapy, apoptosis, radical oxidative species (ROS), protamine deficiency, and smoking (4).

Cyclophosphamide (CP) as a cytotoxic alkylating agent, is one of the most aggressive chemotherapeutic agents, which extensively is used as an antineoplastic drug for the treatment of various cancers, as well as an immunosuppressive agent for organ transplantation, systemic lupus erythematosus and other benign diseases (5, 6). Despite of its wide range of clinical usages, it may produce toxic side-effects in multiple organ systems including the testes in which can lead to male infertility in both animals and human (7). The two CP active metabolites, phosphoramide mustard and acrolein (5), are functional alkylating agents and can potentially produce single strands of DNA-DNA or DNA-protein cross-links (6). In the case of chronic application of toxic anti-cancer drugs, it is and accountable issue to revise their effects and side effects continuously. 

It seems that administration of antioxidant agents such as ginseng as natural herbal extract could be the appropriate approach to reduce the toxicity of CP because they have minimum or no side effects (8). Ginseng (family: Araliaceae) is a native plant of Asian countries. The most common Ginsengs include: Panax ginseng C.A. Meyer (*Asian ginseng*), Panax pseudo-ginseng (*Japanese ginseng*) and Panax quinquefolius L. (*American ginseng*) (8). The Asian and American ginsengs have used for their tonic, stimulant and aphrodisiac properties but they have different bioactivities and chemical compositions.

In recent years, much attention has focused on this plant for its potential effects on sexual function. It was reported that consumption of Ginseng extract improved the sperm parameters in patients with fertility problems (3). It showed that the pharmacological effects of ginseng roots have been attributed to ginsenoside that is the principle active ingredients of ginseng (9, 10).

In the present study, the toxicity effects of CP on reproductive tract in male rats evaluated. The role of ginseng in minimizing anti-cancer-drug-induced infertility evaluated beside its possible mechanism.

## Materials and methods

**Chemicals**

Cyclophosphamide, 2-thiobarbituric acid (TBA), Ham’sF10, NaHCO_3_, Trypan Blue, Eosin-Y, Nigrosin, Ethanol, Hematoxylin, Paraffin, Carnoy’s fixative (methanol/Acetic acid; 1/3), Glutaraldehyde, Phosphate buffered saline, Acridine orange, Aniline blue, Trichloroacetic acid, n-butanol, ethylenediaminetetraacetic acid (EDTA), hydrogen peroxide, acetic acid, and Phosphate buffer were obtained from Sigma Chemicals. St. *American ginseng* tablets purchased from Xiamen Golden Sun Pharmaceutical Co., Ltd. China.

**Animals**

Specific pathogen free, adult male (4-month of age, weighing 220±30 gr) and female (3-month of age, weighing 180-200 gr) albino rats of Wistar strain fostered in animal center of faculty of Science, Urmia University. The Animals housed singly under standard animal facility conditions (Tm, 24±2^o^C, relative humidity, 55±10% and a 12 hr. light/dark photoperiod starting 8.00 p.m.). The animals had access to a standard chow diet and drinking water *ad libitum* throughout the experimental period (about 4 months). 

**Treatment**

CP and *American ginseng* dissolved in normal saline. The ginseng solution was prepared immediately before use. The rats randomly divided into four groups, seven animals each. Group 1 as control received normal saline by intubation, group 2 received CP (6.1 mg/kg/day) for a period of 50 days by intraperitoneal injection. Group 3 received *American ginseng* (500 mg/kg/day) by intubation. Group 4 received *American ginseng* (500 mg/kg/day) 1 hr. prior to the administration of CP. The subdivisions and dosages were based on our previous work (11). The animals weighted weekly in order to control the drug volume and their general health.

At the end of the treatment period, the animals weighted again. All animals were deeply anaesthetized with ether at the end of treatment, and blood was collected from each animal via decapitation into a sterile sample bottle that containing EDTA. Laparotomy conducted to expose the reproductive organs. Epididyms and testis immediately dissected out and weighted by a precise electronic balance. Left testis fixed in formalin solution 10% for histopathological examinations. The plasma and other testis samples separated and frozen by liquid nitrogen and stored at temperature - 80°C until investigation. The epididymis processed for the following analysis

**Sperm parameters assay on collected samples**

The left epididymis carefully separated from the testis and placed in a Petri dish containing Ham's F10. Caudal part of epididymis minced with scissor to release sperm and then was placed in 5 ml of Ham's F10 medium and incubated in CO_2_ incubator at 37^o^C for 5 min to swim out of sperm from the epididymal tubules. The sperm parameters include in count, motility, viability and morphology of sperms determined by standard approaches. 

**Acridine orange DNA denaturation assay**

Thick smears fixed in Carnoy’s fixative (methanol: acetic acid 1: 3) for at least 2 hr. The slides stained for 5 min and gently rinsed with deionized water. Red and green sperm stained sperms seen under the fluorescent light microscope. 400 sperm cells from each staining protocol were evaluated and graded as normal DNA (green) or damaged DNA (yellow to red) (12).

**Aniline blue chromatin quality assay**

Slides prepared by smearing 5 μl of either a raw or washed semen sample. The slides air-dried and fixed for 30 min in 3% glutaraldehyde in phosphate buffered saline. The smears dried and stained for 5 min in 5% aqueous aniline blue solution (pH=3.5). Sperm heads containing immature nuclear chromatin stain blue and those with mature nuclei do not take up the stain. The percentage of spermatozoa stained with aniline blue was determined by counting 400 spermatozoa per slide under bright field microscope (12).

**Histopathological studies**

A portion of the testis from all the animals fixed in 10% formalin, dehydrated in ethanol and embedded in paraffin, sectioned at 5 microns thickness. The sections were then deparafinized with xylene, rehydrated with alcohol and water. The rehydrated sections stained routinely with haematoxylin and eosin (H&E), for evaluation by light microscopy. The diameters of 20 transversely cut seminiferous tubular ducts (STD) per testis determined using an ocular micrometer in a light microscope and their average calculated. In the same tubules, the epithelial height or seminiferous epithelium (SE) measured from the basement membrane to the surface of the epithelium at two different regions and the mean calculated (9). 

Twenty seminiferous tubules counted, and tubule differentiation index (TDI) and spermiation index determined for each group (13). Moreover, testicular sections from each animal evaluated for quality structural changes such as epithelial sloughing, degenerating Leydig cells, abnormal Sertoli cells, and vacuoles (14-16).

**Biochemical analysis**

**Lipid peroxidation (LPO)**

LPO is one of the most useful determinants of the cellular toxicity. Concentration of LPO in plasma and testis evaluated by measurement of the malondialdehyde (MDA) and other lipid peroxide aldehydes. MDA formation as a final product of the peroxidation of lipids can use as an index of the intensity of oxidative stress. The reaction of MDA with thiobarbituric acid generates a colored product that can be measured optically at 535 nm (17).

**Testosterone concentration**

Testosterone quantified employing chemiluminescence method using a commercially available kit and results expressed as ng TST/dl of plasma. 

**Ethical consideration**

This prospective study reviewed and approved by the animal ethics committee of Urmia University of Medical Sciences and carried out according to the National Institute of Health Guide (NIH) for the care and use of laboratory animals guidelines.

**Statistical analysis**

Statistical analysis performed using GB-Stat statistical software. The data distribution determined by Kolmogorov-Smirnov (K-S) test. The results presented as Mean±SD. Statistical analyses were performed using one-way analysis of variance (ANOVA).The significance of difference between groups was evaluated using Tukey test and p<0.01 were considered to be statistically significant.

## Results

All adult male rats survived after treatment period. CP-treated animals were normal without any unhealthy physical symptom compared to controls.

**Sperm chromatin structure**

The data showed that when rats were exposed to CP, percentage of sperm DNA damage significantly increased (p<0.01) in comparison with the control group, while *American ginseng* could decrease CP-induced DNA damage. Besides, when experimental group compared with the control group, the percentage of mature spermatozoa in CP-administration animals had significantly decreased (p<0.01) but by use of *American ginseng* together with CP, there was significantly improvement in quality of chromatin (Table I). 

**Hormone assay**

The evaluation of hormone assay showed that *American ginseng* treatment could significantly increase plasma testosterone concentration in compression with CP group (p<0.01). On the other hand, co-administration of CP and ginseng could recover CP-induced reduction of plasma testosterone (Table II).

**Biochemical findings**

The data also showed that the content of the MDA in the testis and plasma significantly increased (p<0.01) with CP application but decreased to the level of concentration of the control group with *American ginseng* co-treatment (Table II).

**Histology of testis**

**Figure 1:**
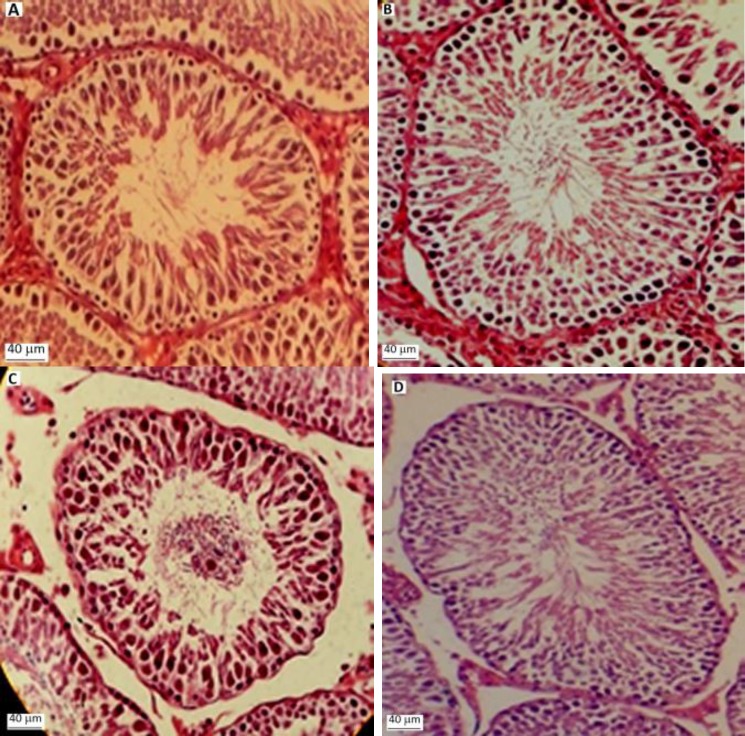
Cross sections of testicular structure in rats treated with CP and/or *American ginseng* (H&E). (A, B) Photomicrographs of testicular section from control and *American ginseng* treated groups’ shows normal feature of the seminiferous epithelium, lumen, Leydig cells and interstitial tissue. (C) A photomicrograph of testicular section from a CP treated rat shows epithelial sloughing and distorted seminiferous epithelium with impaired spermatogenesis. (D) A photomicrograph of a testicular section from a CP + *American ginseng* treated shows nearly normal seminiferous epithelium (×100). Scale bar: 100 µm

The histopathological examination showed the normal testicular structure in control group. In CP treatment group, the diameter (STD) and height (SE) of seminiferous tubules were less than in the control group. However, there were some disturbances in spermatogenesis in the presence of CP although the low number of sperms seen in seminiferous tubules. Not only CP increased the disintegration of epithelial cells in seminiferous tubule like as shedding into the lumen, decomposition of Sertoli cells, widening of the interstitial space and vacuolization in interstitial tissues, but also the atrophy in the most of the tubules of the Leydig cells and dispersion with picnotic nuclei observed. The percent of both TDI and SPI declined in CP treated animals in comparison with control group. Testis sections in rats treated with CP and *American ginseng* showed less damage than those in rats treated with CP only (Table III, Figure 1 A-D).

**Table I:** Effects of CP and *American ginseng* on the sperm DNA and chromatin integrity of male rats

**Sperm parameters (%)**	**Con**	**CP**	**Gin**	**CP+Gin**
DNA damage	15.73 ± 0.64	44.83 ± 2.98 b	9.55 ± 0.68 ab	19.71 ± 2.66^ a^
Immature chromatin	2.5 ± 0.64	18.75 ± 1.75 b	2 ± 0.40 a	8 ± 1.08 ab

Data presented as Mean±SD. The significance difference between groups was evaluated using Tukey test.

Con: control; CP: cyclophosphamide; Gin: ginseng.

a = significant different from cyclophosphamide group.

b = significant different from control groups (p<0.01).

**Table II:** Effects of CP and *American ginseng* on the oxidative stress biomarkers in plasma and testis and testosterone concentration in male rats

**Variables **	**Con**	**CP**	**Gin**	**CP + Gin**
Plasma lipid peroxidation (μmol/ml)	0.154 ± 0.01	1.133 ± 0.08 b	0.054 ± 0.03 ab	0.513 ± 0.02 a
Testis lipid peroxidation (μmol/ml)	0.081 ± 0.03	1.135 ± 0.08 b	0.027 ± 0.01 a	0.062 ± 0.02 a
Testosterone (ng/dl)	2.25 ± 0.149	0.70 ± 0.37 b	2.77 ± 0.12 a	1.89 ± 0.21 a

The values are expressed as Mean±SD

Con: control; CP: cyclophosphamide; Gin: ginseng.

a = significant different from cyclophosphamide groups.

b = significant different from control groups (p<0.01).

**Table III:** The effects of CP and *American ginseng* on the testis structure

**Variables **	**Con**	**CP**	**Gin**	**CP + Gin**
TDI (%)	92.85 ± 2.85	61.42 ± 3.40 ab	98.57 ± 1.42 a	84.28 ± 3.68 a
SPI (%)	90 ± 3.08	48.57 ± 3.40 b	97.14 ± 1.84 a	82.85 ± 3.59 ab
STD (μm)	2695.29 ± 121.74	2100.95 ± 104.15^ b^	3014.66 ± 224.21^ a^	2774.06 ± 106.83 a
SE (μm)	525 ± 52.44	307.85 ± 25.44 b	571.83 ± 99.51 a	543.07 ± 93.58 a

The values are expressed as Mean±SD.

Con: control; CP: cyclophosphamide; Gin: Ginseng. TDI: Tubule differentiation index; SPI: spermiation index; STD: Seminiferous diameter; SE: Seminiferous height.

a =significant different from CP groups.

b =significant different from control groups (p<0.01).

## Discussion

Cancer pharmacotherapy involved in using some anticancer drugs with side effects. These can be use in an effective period but the side effects make some limitations. These drugs have associated with vary degrees of reproductive impairment. These side effects may be temporary or permanent (3). The CP as a common anticancer agent has a broad spectrum of antitumor activity in the treatment of human cancer. The duration and amount of use of anticancer agent can produce some side effects that the recovery of these changes are dependent on the type and cumulated dose beside the duration of the exposure (18). 

The alleviating of the side effects is a well-known parallel therapy in addition anticancer drug use. Animal models provide a very good alternative for humans to carry out the toxicological study of chemotherapy drugs (19). In the current study, it showed that the application of CP for 50 days increased sperm DNA and chromatin damages that confirming a previous report (20). Additionally, the chronic application of CP that used in the present study caused histological changes in the seminiferous tubules. These results coincide with the findings of a previous study (21). 

Currently, there are no effective clinical approach to avoid these toxic side effects. Herbal extract for pharmacotherapy may be act as an effective proposal to treat infertility. Ginseng has also used to improve sex performance and satisfaction since many years ago. Several studies have reported that ginseng extracts may have stimulatory effects on spermatogenesis, DNA and protein syntheses. Ginseng has shown to improve the sperm parameters. The meta-analysis studies of randomized clinical trials confirmed these findings also (22-25).

Although, the responsible mechanisms for CP-induced reproductive toxicity is correctly unknown; however numerous studies have shown that oxidative stress, lipid peroxidation and apoptosis are the main candidates (5, 26). Previous studies showed that acrolein interferes with the tissue antioxidant defense system, and with inactivation of microsomal enzymes, increase reactive oxygen free-radicals and lipid peroxidation (27).

Concentration of lipid peroxidation evaluated with measurement of malondialdehyde. In this study, biochemical evaluation of testes and plasma showed that exposure to CP increased MDA concentration. A previous study reported that ginseng extracts exhibited protective effects against peroxidation of unsaturated fatty acid (28).

Primakoff and Myles showed that in the case of ROS level elevation, spermatozoa will undergo lipid peroxidation (29). Increased ROS generation and reduced antioxidant capacity have negatively correlated with sperm concentration and sperm nuclear DNA integrity. The declination of sperm concentration can cause by the elimination of sperm cells at different stages of development, atrophy of Leydig cells and a significantly lower rate of spermatogenesis. Peroxidation of sperm lipids and generation of ROS decompose the structure of the lipid matrix in the membranes of spermatozoa, presumably by a rapid loss of intracellular ATP leading to damage in sperm flagellum and axonemal. The mentioned maladaptation has strong negative effects on the vital functions of the sperms such as motility (30, 31).

Another possible reason for the CP cytotoxic effect is DNA damage induced by its main metabolic product, phosphoramide mustard (32). The existence of morphologic abnormalities such as cytoplasmic droplets have associated with ROS production. These abnormalities are sign of sperm immaturity, which positively correlated with sperm DNA damage (33). All of these reasons have considered as the major effects of male infertility. 

We found a statistically significant decrease in testosterone concentration in CP-treated animals. This result is in agreement to a previous finding that CP treatment led to decrease in the levels of testosterone in mice (19). Reduction in plasma testosterone to be resulting from toxic effect of drug on the Leydig cells that confirmed by our histological findings. Chemotherapy may have either a direct toxic effect on the Leydig cells or indirect via germinal epithelial damages can lead malfunction of the Leydig cells. In addition, reduction in testosterone concentration may impair epididymal function (34). Chromatin in mammalian spermatozoa constructed in a unique pattern. An usual abnormality of sperm chromatin is due to deficiency of sperm protamines (7). 

The inter- and intra-molecular disulfide cross-links between the cysteine-rich protamines are responsible for the compaction of the sperm nucleus, which is important to protect the sperm genome from external stresses (35). Acridine orange staining demonstrated that sperm DNA damage increased by CP administration. Although low levels of ROS in semen are important for normal sperm function, the high levels of ROS can adversely can affect sperm functions and sperm DNA integrity (36) that associate with fertilization, embryo cleavage and pregnancy rates (24). 

The rapidly dividing germinal epithelium of the testis is a natural target for cytotoxic products. The results of the present study indicated that CP administration at the dose of 6.1 mg/kg resulted in damage to Sertoli, Leydig cell populations as well as abnormality in sperm morphology. The decreases in diameter (STD) and height (SE) of the seminiferous tubule in the presence of CP occurred. The reduced seminiferous tubule diameter reflects tubular shrinkage, which may occur due to sloughing of epithelial cells. Because of Sertoli cells are supportive cells of the seminiferous epithelium, they are key roles in regulating spermatogenesis. Abnormality in the formation and development of Sertoli cells results in the loss of their ability to support germ cell survival. CP- induced histological changes in the seminiferous tubules resulting in the suppression of spermatogenesis and induced abnormalities in spermatozoa. All these factors may contribute to male infertility by reducing sperm function. 

Ginseng root aqueous extract shows cytoprotective effects in mouse testes, which mediated by decreasing the intracellular production of hydrogen peroxide (5). In summary, a balance between the reactive oxygen species and tissue antioxidant capacity appears to be necessary for survival and normal functioning of spermatozoa.

## Conclusion

This study demonstrates that CP treatment induces significant oxidative stress, which is severely impaired function and structure of testis and *American ginseng* has protective and therapeutic effects on CP-induced toxicity. To date, studies have reported that Ginseng has an antioxidant effect against cellular oxidative damage in tissues. Probably, its antioxidant properties originate from its ability to scavenging of free radicals and suppression of DNA oxidative damage. These properties of the ginseng may provide many beneficial effects against organ damages. Therefore, *American ginseng* as a preventive agent decrease the side effects of CP. These results might help in recovery from reproductive gonads dysfunction in cancer patients that have been received CP.

## Note

***This article extracted of M.Sc. Thesis. (Akram Hosseini)***
